# In Vitro Evaluation of Pharmacokinetic Properties of Selected Dual COX-2 and 5-LOX Inhibitors

**DOI:** 10.3390/ph17101329

**Published:** 2024-10-05

**Authors:** Jelena Bošković, Vladimir Dobričić, Jelena Savić, Jelena Rupar, Mara Aleksić, Bojan Marković, Olivera Čudina

**Affiliations:** 1 Department of Pharmaceutical Chemistry, University of Belgrade–Faculty of Pharmacy, Vojvode Stepe 450, 11000 Belgrade, Serbiabojan.markovic@pharmacy.bg.ac.rs (B.M.);; 2 Department of Physical Chemistry and Instrumental Methods, University of Belgrade–Faculty of Pharmacy, Vojvode Stepe 450, 11000 Belgrade, Serbia

**Keywords:** N-hydroxyurea and hydroxamic acid, dual COX-2 and 5-LOX inhibitors, passive gastrointestinal absorption, HSA binding affinity, microsomal stability

## Abstract

Evaluation of pharmacokinetic properties is a significant step at the early stages of drug development. In this study, an in vitro evaluation of the pharmacokinetic properties of five newly synthesized compounds was performed. These compounds belong to N-hydroxyurea and hydroxamic acid derivatives and analogs of NSAIDs indomethacin, flurbiprofen, diclofenac, ibuprofen, and naproxen (compounds **1**, **2**, **3**, **11,** and **12**, respectively) with dual COX-2 and 5-LOX inhibitory activity. Two in vitro methods (biopartitioning micellar chromatography (BMC) and PAMPA) were used to evaluate passive gastrointestinal absorption, while high-performance affinity chromatography (HPAC) and differential pulse voltammetry (DPV) were used to evaluate binding to human serum albumin (HSA). The introduction of N-hydroxyurea and hydroxamic acid groups into the structure of NSAIDs decreases both expected passive gastrointestinal absorption (BMC **k** values were from 3.02 to 9.50, while for NSAIDs were from 5.29 to 13.36; PAMPA **–logPe** values were between 3.81 and 4.76, while for NSAIDs were ≤3.46) and HSA binding (HPAC **logk** values were from 2.03 to 9.54, while for NSAIDs were ≥11.03; DPV peak potential shifts were between 7 and 34, while for NSAIDs were ≥54). Structural modifications of all tested compounds that increase lipophilicity could be considered to enhance their passive gastrointestinal absorption. Considering lower expected HSA binding and higher lipophilicity of tested compounds compared to corresponding NSAIDs, it can be expected that the volume of distribution of compounds **1**, **2**, **3**, **11,** and **12** will be higher. Reduced HSA binding may also decrease interactions with other drugs in comparison to corresponding NSAIDs. All tested compounds showed significant microsomal instability (25.07–58.44% decrease in concentration) in comparison to indomethacin (14.47%) and diclofenac (20.99%).

## 1. Introduction

The side effects of COX-2 inhibitors may arise from the potentiation of arachidonic acid oxidation by the 5-LOX enzyme. Therefore, dual inhibition of COX-2 and 5-LOX enzymes can provide a better anti-inflammatory effect with fewer side effects [1]. One strategy in the development of dual COX-2 and 5-LOX inhibitors involved the modification of conventional nonsteroidal anti-inflammatory drugs (NSAIDs) [2,3]. The obtained results indicated that the replacement of the carboxyl group in the NSAID structure with a hydroxamic acid group leads to significant 5-LOX inhibitory activity [4]. The integration of COX-2 and 5-LOX pharmacophores has resulted in the development of various dual inhibitors, among which tepoxalin is particularly noteworthy [5,6,7]. Derivatives of di-tert-butylphenol emerged as potent anti-inflammatory agents. Tebufelone demonstrated inhibition of both prostaglandin and leukotriene synthesis [8]. Additionally, the di-tert-butylphenol derivative darbufelone showed selective and potent inhibition of COX-2 and 5-LOX enzymes [9]. Several 3-aryl isocoumarin derivatives emerged as promising agents for combating inflammatory diseases and cancer, given that isocoumarins are natural compounds with a broad spectrum of biological activities. 3-aryl isocoumarins exhibited strong inhibitory effects on 5-LOX in vitro and on PGE2 in vivo in HeLa cells [10].

Replacing the carboxyl functional group of nonsteroidal anti-inflammatory drugs (NSAIDs) with N-hydroxyurea and hydroxamic acid functional groups is believed to result in the expression of 5-LOX inhibitory activity, while COX-2 inhibitory activity may be lost or retained. Several N-hydroxyurea and hydroxamic acid derivatives and analogs of conventional NSAIDs were synthesized, and their in vitro enzymatic inhibitory activities were assessed. The conventional NSAIDs did not inhibit the 5-LOX enzyme, whereas their N-hydroxyurea and hydroxamic acid derivatives and analogs demonstrated significant 5-LOX inhibitory activity [3,4].

**Figure 1:**
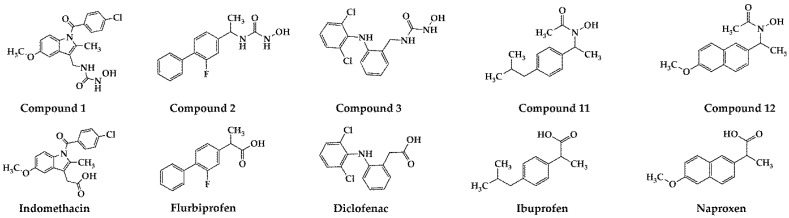
Synthesized compounds **1**, **2**, **3**, **11**, **12**, and commercially available NSAIDs (indomethacin, flurbiprofen, diclofenac, ibuprofen, and naproxen).

**Scheme 1:**
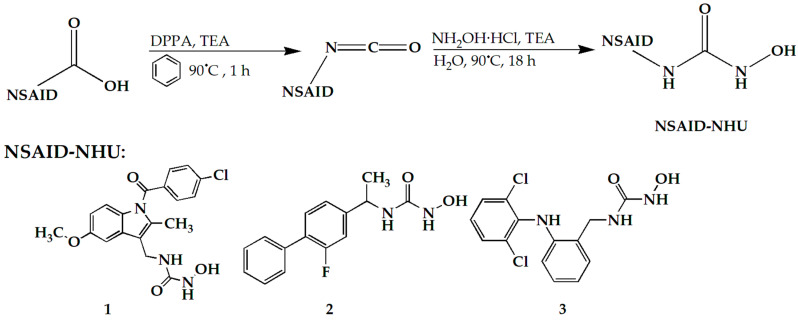
Synthesis steps of compounds **1**, **2**, and **3**.

**Scheme 2:**
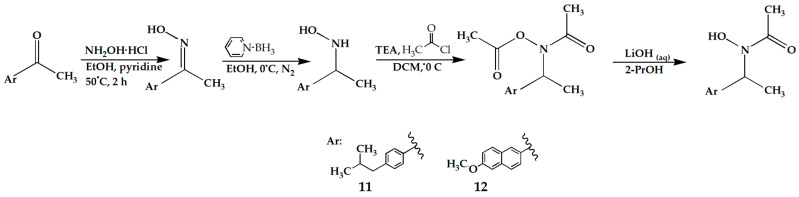
Synthesis steps of compounds **11** and **12**.

In our previously published study [11], compounds **1**, **2, 3, 11,** and **12** were synthesized by replacing the carboxyl functional group of five NSAIDs (indomethacin, flurbiprofen, diclofenac, ibuprofen, and naproxen, respectively) with N-hydroxyurea and hydroxamic acid functional groups (Figure 1 and Scheme 1 and Scheme 2). These NSAIDs are non-selective COX-1 and COX-2 inhibitors, used in therapy as anti-inflammatory drugs. Synthesized compounds **1**, **2, 3, 11**, and **12** proved to be the best dual COX-2 and 5-LOX inhibitors presented in this study. The obtained IC_50_ values were in the range of 5.26–83.42 µM for COX-2 inhibition and in the range of 1.04–5.71 µM for 5-LOX inhibition. In addition, weak COX-1 inhibition was observed for all of them (IC_50_ > 100 µM), indicating good COX-2/COX-1 selectivity. Since inhibition of both COX-2 and 5-LOX enzymes is known to be a significant strategy in the treatment of colorectal and pancreatic cancers [12,13,14], compounds **1**, **2**, **3**, **11**, and **12** were further evaluated as cytotoxic agents. Compounds with the best cytotoxic activity against tested cancer cell lines were **1**, **2**, and **3**. These compounds also showed good antimigratory effects, while compound **3** did not show toxic effects on zebrafish embryos at therapeutic doses [15].

Although good potency and selectivity are the main criteria for the selection of a drug candidate, they do not guarantee good in vivo efficacy because of inadequate pharmacokinetic properties (poor absorption, high metabolic rate, and low in vivo free concentration when the drug candidate binds to plasma proteins in high degree). The estimation of pharmacokinetic properties is very important at early stages of drug development in order to reduce drug candidate failure in later development stages [16]. Pharmacokinetics employs mathematical models to describe what happens to a chemical substance within the body, and the effects of a drug are strongly related to its concentrations in the target tissues. Variations in drug concentration over time are influenced by the rates at which the body absorbs, distributes, metabolizes, and excretes the drug.

Absorption is the process through which a drug enters the body, specifically the systemic circulation. Various obstacles can hinder drug molecules from reaching the bloodstream, depending on how the drug is administered. Oral drug administration is one of the most common and the most preferable methods of drug administration [17]. Maximizing the bioavailability of orally administered drugs is a key goal for the pharmaceutical industry during new drug development. For oral administration, the amount of drug that ultimately reaches systemic circulation depends on how much is absorbed by the intestinal epithelium. Additionally, several other factors can influence bioavailability [18]. Various in vitro techniques have been developed to assess drug absorption rates, extent, and mechanisms. Cultured cell systems, such as Caco-2 cells, are widely used due to their moderate complexity and potential as screening models [19]. The PAMPA test (Parallel Artificial Membrane Permeability Assay) and biopartitioning micellar chromatography (BMC) represent fast and simple in vitro methods for the prediction of passive gastrointestinal absorption. In BMC, surfactants are used above their critical micelle concentration to create micelles that, along with surfactant monomers, alter the stationary phase to mimic the cell membrane. BMC is used in studying compound absorption through the gastrointestinal tract, skin, and blood–brain barrier and for predicting toxicity [20,21,22]. BMC is valued for its reproducibility, cost-effectiveness, and flexibility in selecting stationary phases and surfactants [20]. The PAMPA test assesses compounds’ permeability through an artificial membrane that mimics physiological membranes. Artificial membranes can be formed using hydrophobic or hydrophilic filters and various lipid and solvent combinations. PAMPA evaluates passive diffusion, as active transport cannot be simulated. This is a minor limitation, since about 95% of known drugs are absorbed via passive diffusion. PAMPA is increasingly favored for its cost-effectiveness in early ADME screening [23,24]. PAMPA simulates the transport of compounds across various biological membranes, including the gastrointestinal tract, skin, and blood–brain barrier [25,26,27].

A part of the administered dose of a drug in many cases is reversibly bound to proteins in the human blood and, therefore, is not available for interaction at the target site, while the unbound portion is referred to as the free drug concentration. The bound portion acts as a depot of the free drug and can affect drug pharmacokinetics and pharmacodynamics, as well as toxicity. Human serum albumin (HSA) is the most abundant soluble protein in human blood plasma, making up to 65% of the total plasma protein content in the human organism [28]. HSA has an important physiological role in maintaining osmotic pressure, transporting hormones, fatty acids, and other compounds, as well as buffering the pH of the blood [29]. The interaction of drugs with HSA greatly affects the volume of distribution and the rate of their elimination [30]. In some pathological conditions, the concentration or properties of HSA are altered. Because of this, the interaction of a drug with HSA is altered, which affects the free drug concentration and subsequently the drug’s pharmacokinetics [31]. The estimation of HSA binding is very useful at the early stages of drug research, and it can be performed using various methods, such as high-performance affinity chromatography (HPAC) and electrochemical methods. The specificity of HPAC is the column, which consists of immobilized HSA, and the advantages include short time of analysis, good precision, and possibility of automation [32]. Electrochemical methods are used to investigate interactions between HSA and a potential drug. The usual and most common approach is to study and analyze the change in the position and intensity of the investigated drug electrochemical signal after the interaction with HSA [33,34,35,36,37]. When the mentioned parameters change, the existence of an interaction is confirmed, and the type of interaction can be assumed. These methods can also be used to determine the percentage of bound drug to HSA, the binding constant, and the stoichiometry of the formed complex and are characterized by versatility, high sensitivity, and simplicity. They are inexpensive and provide rapid results [33].

Drug metabolism investigation plays an important role in drug discovery, and the effects of drug metabolism on pharmacokinetics, pharmacodynamics, and safety should be carefully considered. Drug metabolism is a complex biotransformation process where drugs are transformed to metabolites by various metabolizing enzymes. It consists of two phases: the 1st phase (oxidation, reduction, and hydrolysis) and the 2nd phase (conjugation reactions). The main site of drug metabolism is the liver. Liver microsomes are subcellular particles derived from the endoplasmic reticulum of hepatic cells and contain numerous drug metabolizing enzymes. Microsome pools from various sources are useful in the study of xenobiotic metabolism and drug interactions. In drug discovery, microsomes of human or animal origin can be used [38].

The aim of this study was to evaluate selected pharmacokinetic properties (passive gastrointestinal absorption, binding to HSA, and microsomal stability) of compounds **1**, **2**, **3**, **11**, and **12** as the best dual COX-2 and 5-LOX inhibitors synthesized in our laboratory. For that purpose, two in vitro methods were used for the evaluation of passive gastrointestinal absorption (BMC and PAMPA), and also two in vitro methods were used for the evaluation of HSA binding (HPAC and differential pulse voltammetry (DPV)). Incubation with CD-1 mouse liver microsomes was performed in order to test microsomal stability.

## 2. Results and Discussion

### 2.1. Evaluation of Gastrointestinal Absorption

Biopartitioning micellar chromatography (BMC) and Parallel Artificial Membrane Assay (PAMPA) were used to evaluate passive gastrointestinal absorption of compounds **1**, **2**, **3**, **11**, and **12**. Commercially available nonsteroidal anti-inflammatory drugs acting as COX inhibitors (indomethacin, flurbiprofen, diclofenac, ibuprofen, and naproxen) were used as standards for comparison. These standards are most commonly administered orally.

#### 2.1.1. BMC

BMC was performed using polyoxyethylene lauryl ether (Brij 35) as the most frequently used surfactant in this method. All other parameters (pH, temperature, and salinity) were set to achieve physiological conditions. The retention of compounds is mostly influenced by their lipophilicity and their interactions with both the modified stationary phase and the micelles in the mobile phase [22,39]. Therefore, the elution behavior is determined by the combined effects of micelles, the stationary phase, and water [40,41]. An important factor for this method is finding the optimal balance between organic solvent and aqueous phase [41,42].

Retention factors (**k**) of compounds **1**, **2**, **3**, **11**, and **12** and corresponding standards are presented in Table 1.

The obtained **k** values of compounds **1**, **2**, **3**, **11**, and **12** were in the range from 3.02 to 9.50. The **k** values of compounds **1**, **2**, **11**, and **12** were lower in comparison to corresponding parent NSAIDs (indomethacin, flurbiprofen, ibuprofen, and naproxen, respectively), so lower passive gastrointestinal absorption is expected compared to them (calculated *p* values were lower than 0.05 for each pair). Compound **3** had a slightly higher **k** value compared to its parent NSAID diclofenac (9.33 vs. 8.25).

#### 2.1.2. PAMPA Test

The PAMPA test uses a 96-well plate coated with an impregnated filter material, forming an artificial membrane that separates donor and acceptor compartments [24]. Egg lecithin in dodecane is commonly used to simulate membrane lipids, while hexadecane is effective for mimicking gastrointestinal membranes. The latter model is characterized by high reproducibility, shorter incubation time (5 h vs. 17 h), and the possibility to calculate membrane retention, which can significantly affect permeability coefficients. Therefore, hexadecane dissolved in hexane was chosen in this study.

The PAMPA permeability results are presented in Table 2. **Pe** is the effective passive permeability coefficient. For simplicity, the parameter **–logPe** was used in further discussion, and a higher **–logPe** value indicates lower passive gastrointestinal absorption. Membrane retention (**R**) of all compounds (including standards) was low (<10%), except for compound **11**, which showed significant retention (24%).

The **–logPe** values of compounds **1**, **2**, **3**, **11**, and **12** were in the range from 3.81 to 4.76. The **–logPe** values of tested NSAIDs (indomethacin, flurbiprofen, diclofenac, ibuprofen, and naproxen) were ≤3.46. Therefore, lower passive gastrointestinal absorption is expected for compounds **1**, **2**, **3**, **11**, and **12** in comparison to all parent NSAIDs (*p* value calculated taking into consideration the standard with the highest **–logPe** (naproxen, 3.46) and the tested compound with the lowest **–logPe** (compound **12**, 3.81) was 0.016).

All standards tested in this study (indomethacin, flurbiprofen, diclofenac, ibuprofen, and naproxen) belong to BCS (Biopharmaceutical Classification System) class II (high permeability and low solubility), and results that we obtained using PAMPA are in agreement with this [43]. In our laboratory, using the same PAMPA experimental setup, two other commercially available drugs were also tested (unpublished material): acyclovir (BCS class III—low permeability and high solubility) and aripiprazole (BCS class IV—low permeability and low solubility). The PAMPA permeability coefficients (aripiprazole: 4.38 ± 0.05; acyclovir: 4.51 ± 0.08) indicate low permeability, which is also in accordance with the BCS classification. These results confirm the validity of the performed PAMPA assay.

#### 2.1.3. Comparison of the Results of Two Methods and Discussion on Passive Gastrointestinal Absorption Potential

The results of the BMC and PAMPA tests were quite consistent. Compounds **1**, **2**, **11**, and **12** should have lower passive gastrointestinal absorption than corresponding NSAIDs (indomethacin, flurbiprofen, ibuprofen, and naproxen, respectively). The only difference was noticed in the case of compound **3**, for which two methods give different conclusions. Generally, it can be concluded that the introduction of N-hydroxyurea and hydroxamic acid functional groups should result in a significant decrease in passive gastrointestinal absorption. Therefore, structural modifications of compounds **1**, **2**, **3**, **11**, and **12** that increase lipophilicity (such as addition of aromatic rings or alkylation of the nitrogen in the N-hydroxyurea group) could be considered to enhance their passive gastrointestinal absorption.

### 2.2. Evaluation of HSA Binding

HSA possesses several binding sites, so it can interact with a wide range of structurally diverse drugs. Some of the drug classes, such as NSAIDs, are bound to HSA in a very high percentage (up to 99%) [44]. These drugs may increase the free concentration of coadministered drugs by their displacing from complex with HSA. One of the well-known examples of this type is the interaction of NSAIDs with warfarin, a drug with a narrow therapeutic index. The consequence of this interaction is increased free concentration of warfarin, causing increased bleeding [45].

In this study, two different methods (HPAC and DPV) were used to examine the interaction between HSA and compounds **1**, **2**, **3**, **11**, and **12**. The same standards as in the evaluation of the passive gastrointestinal absorption were used (indomethacin, flurbiprofen, diclofenac, ibuprofen, and naproxen).

#### 2.2.1. High-Performance Affinity Chromatography (HPAC)

Recently, interactions of many newly synthesized structurally diverse compounds with HSA were successfully studied using this technique [46,47,48]. The degree of binding of the tested compounds to HSA was evaluated based on their retention behavior on the stationary phase modified with HSA. The logarithmic values of retention factors (**logk**) were used to evaluate indirectly and compare the drug–HSA interaction. The obtained results are presented in Table 3.

The **logk** values of compounds **1**, **2**, **3**, **11**, and **12** were in the range of 2.03 to 9.54. **logk** values of indomethacin, flurbiprofen, diclofenac, ibuprofen, and naproxen were significantly higher (*p* value calculated taking into consideration compound with the highest **logk** (compound **1**, 9.54) and standard with the lowest **logk** (flurbiprofen, 11.03) was 0.048), which indicates stronger interaction with HSA (**logk** values of indomethacin, diclofenac, ibuprofen, and naproxen were higher than 70, indicating the strongest HSA interactions).

#### 2.2.2. Differential Pulse Voltammetry (DPV)

**Figure 2:**
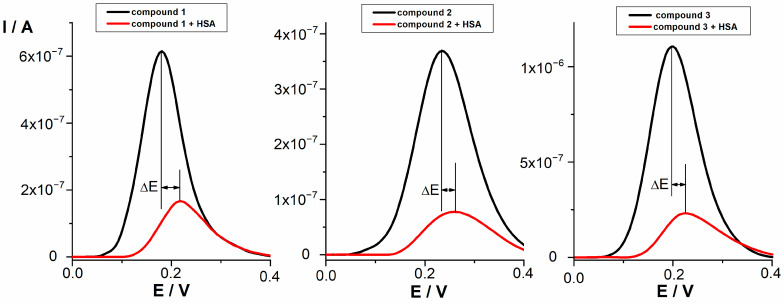
DPV voltammograms of compounds **1**, **2**, and **3** (0.05 mmol/L) before and after interaction with HSA (0.01 mmol/L).

The electrochemical oxidation of the tested compounds at a concentration of 0.05 mmol/L in PBS buffer pH 7.4 was investigated by differential pulse voltammetry (DPV). DPV voltammograms of selected compounds are presented in Figure 2. According to the shape of the peak, the half peak width (W_1/2_ ~ 100−140 mV), and additional cyclovoltammetric measurements, the oxidation process proceeds as irreversible with the transfer of one electron. HSA showed no oxidation or reduction peak under the given conditions. For all tested compounds, a preliminary study of the interaction with HSA was performed at a concentration of 0.05 mmol/L and different HSA concentrations (0.05–10 μmol/L) using DPV. The same procedure was used to study the interaction between NSAIDs (indomethacin, diclofenac, and naproxen) and HSA. Flurbiprofen and ibuprofen proved to be electrochemically inactive under the experimental conditions used. It was observed that the peak potentials of all tested compounds shifted towards more positive potentials in the presence of HSA. At the same time, the peak current decreased. It has been reported that the shift in the potential of an electroactive ligand is a suitable criterion to determine the mode of the macromolecule–ligand interaction. In this context, a positive shift in potential is an indication of hydrophobic interaction of investigated compounds with HSA [34]. The decrease in peak current could be due to the formation of a non-electroactive supramolecular compound by the drug–protein interaction, which reduces the concentration of free drug. This behavior suggests that tested compounds and HSA form a non-electroactive complex [49]. The shift in peaks potential (ΔE) and percentage of bound drug after interaction with HSA for all tested compounds are presented in Table 4.

NSAIDs with a good ability to bind to HSA (indomethacin, diclofenac, and naproxen) showed the shift of 70 mV, 54 mV, and 168 mV, respectively. All tested compounds showed lower shifts in comparison to NSAIDs (*p* value calculated taking into consideration compound with the highest shift (compound **1**, 34) and standard with the lowest shift (diclofenac, 54) was 0.004). Among tested compounds, the strongest change in the position of the oxidation peak after interaction was observed for compound **1** (ΔE = 34 mV) and followed by very similar shifts for compounds **2** and **3**, indicating that the interaction with HSA exists and can be characterized as hydrophobic. Compounds **11** and **12** displayed a smaller change in the peak position, indicating slightly weaker hydrophobic interaction. The decrease in current intensity was observed for all compounds as a result of the existence of the interactions with HSA. The percentage of bound drug for indomethacin, naproxen, and diclofenac (94.3–80.5%) indicates stronger interaction with HSA compared to lower values (80.2–70.4%) obtained for compounds **1**, **2**, **3**, **11**, and **12**. Since no new oxidation peaks occurred after the interaction, the observed decrease in peak current and the percentage of bound drug indicate that a non-electroactive supramolecular compound was formed as the consequence of the all tested compounds–HSA interactions.

#### 2.2.3. Comparison of the Results of Two Methods and Discussion on Distribution Properties

The results of both methods were quite consistent. Results for compounds **1**, **2**, **3**, **11**, and **12** indicate that the introduction of N-hydroxyurea and hydroxamic acid groups reduces binding affinity to HSA, compared to the parent NSAIDs. Consequently, compounds **1**, **2**, **3**, **11**, and **12** may potentially interact less with other drugs compared to standard NSAIDs. Among tested compounds, both methods indicate that **1**, **2**, and **3** should bind more strongly to HSA than **11** and **12**. Many NSAIDs are acidic, and their ionization levels vary with pH. Evoli et al. investigated the ability of commercially available NSAID ibuprofen to bind HSA in both ionized and unionized forms. They proved that ibuprofen in ionized form showed much greater affinity for HSA than in unionized form. Interactions between the carboxylate group of ibuprofen and positively charged (or polar) protein residues contributed significantly to the binding [50]. Compounds **1**, **2**, **3**, **11**, and **12** are less acidic than NSAIDs (according to the Marvin Sketch 23.14.0 [51] prediction, unionized forms of compounds **1**, **2**, **3**, **11**, and **12** are dominant in the whole range of physiologically important pH values, i.e., at 2 < pH ≤ 7.4), so this might be one of the reasons for their lower affinity to HSA in comparison to tested NSAIDs. Since HSA binding and membrane/lipid partition are the major driving forces for a drug’s distribution into the body [52], volume of distribution can be predicted by taking into consideration the drug’s lipophilicity and HSA binding properties. Drugs with higher lipophilicity have a higher chance of leaving the plasma. According to MarvinSketch 23.14.0 [51], predicted logD values at pH 7.4 (logD_7.4_) of compounds **1**, **2**, **3**, **11**, and **12** (2.50, 2.79, 3.23, 3.01, and 2.15, respectively) were higher than logD_7.4_ values of corresponding NSAIDs indomethacin, flurbiprofen, diclofenac, ibuprofen, and naproxen (0.62, 1.69, 1.10, 1.34, and -0.02, respectively). Considering lower HSA binding and higher lipophilicity of compounds **1**, **2**, **3**, **11**, and **12** compared to corresponding NSAIDs, it can be expected that the volume of distribution of compounds **1**, **2**, **3**, **11**, and **12** will be higher.

If one drug has altered HSA binding compared to another one, this might affect the rate of its metabolism since the higher amount of the drug will be more accessible to enzymes. The effect of HSA binding on drug clearance is dependent on its major route of elimination. In the case of renally cleared drugs, HSA binding has a strong effect since only the unbound drug fraction can be excreted in this way. In the case of hepatically excreted drugs, the degree of HSA binding does not play an important role [53]. However, the assessment of how lower HSA binding of compounds tested in this study impacts their metabolism rate and clearance requires additional in vivo experiments.

### 2.3. Stability in Acidic Medium and Solubility

In order to test gastric stability, compounds were incubated in 0.01 M HCl (pH = 2) for 2 h. Moreover, 0.01 M HCl was chosen because this acid is naturally present in the stomach and simulates its pH (between 1 and 3), while incubation time (2 h) corresponds to the average retention of drugs in the stomach after an oral administration. Peak areas at the beginning (t_0_) and at the end of the incubation (t) and the decrease in peak areas between these two time points are presented in Table 5.

The decreases in peak areas were less than 2.33, which indicates good stability in acidic medium and also that good stability in the stomach could be expected after oral administration of tested compounds (the only statistically significant decrease in peak area was observed for compound **3**, and it was 2.33%).

As discussed in the previous section, all tested compounds are dominantly unionized in the whole range of physiologically important pH values (2 < pH ≤ 7.4). Therefore, water was used for the determination of solubility. Solubility of tested compounds was 1.5 mg/L (compound **1**), 14.28 mg/L (compound **2**), 5.45 mg/L (compound **3**), 19.35 mg/L (compound **11**), and 11.76 mg/L (compound **12**). According to USP34, all tested compounds can be defined as practically insoluble since their solubilities are ≤100 mg/L [54].

### 2.4. Microsomal Stability

Microsomal stability (and thus susceptibility to metabolism) was tested using microsomes derived from CD-1 mouse liver. According to the applied protocol, both phases of metabolism were activated by the addition of cofactors NADPH and UDP-GlcUA. Propranolol (an antihypertensive drug) was used as the positive control, while NSAIDs indomethacin and diclofenac were used as standards for comparison. Due to poor ionization in the mass spectrometer, the other three NSAIDs (flurbiprofen, ibuprofen, and naproxen) could not be reliably detected and quantified. Results are presented in Table 6.

The highest decrease in peak area and the lowest microsomal stability were observed for compounds **1** (53.98%), **3** (55.93%), and **12** (58.44%). The lowest decrease in peak area and the highest microsomal stability were observed for compound **11** (25.07%). All tested compounds showed lower microsomal stability than indomethacin (14.47%) and diclofenac (20.99%), while microsomal stability of the positive control propranolol (42.03%) was within the range of values of compounds **1**, **2**, **3**, **11**, and **12**. It can be expected that compounds **1**, **3**, and **12** will undergo hepatic metabolism in a higher extent than compounds **2** and **11** and tested NSAIDs.

## 3. Materials and Methods

### 3.1. Chemicals

Compounds **1**, **2**, **3**, **11**, and **12** were synthesized as previously described [11]. Human serum albumin (HSA), diclofenac, Brij 35, disodium hydrogen phosphate (Na_2_HPO_4_), sodium dihydrogen phosphate monohydrate (NaH_2_PO_4_ × H_2_O), *ortho*-phosphoric acid (85%), potassium dihydrogen phosphate (KH_2_PO_4_), potassium chloride (KCl), sodium chloride (NaCl), 0.1 M hydrochloric acid, microsomes from liver (pooled, from mouse (CD-1) male, catalogue number: M9441), NADPH tetrasodium salt, alamethicin from Trichoderma viride, and uridine 5′-diphosphoglucuronic acid trisodium salt (UDP-GlcUA) were purchased from Sigma-Aldrich (St. Louis, MO, USA). Indomethacin, dimethyl sulfoxide (DMSO), hexane, ammonium acetate, acetonitrile HPLC purity, and methanol HPLC purity were purchased from Fisher Scientific (Loughborough, UK). Flurbiprofen and hexadecane were purchased from Acros Organics (Geel, Belgium). Deionized water (TKA water purification system, Niederelbert, Germany) was used throughout this study.

### 3.2. Biopartitioning Micellar Chromatography (BMC)

BMC was performed on an Agilent 1200 Series (Agilent Technologies, Santa Clara, CA, USA) supplied with a binary pump, manual injector (20 µL), and DAD detector (190–950 nm) using a C18 column (ZORBAX Extend-C18 Analytical 4.6 × 150 mm; 5 µm, Agilent Technologies, Santa Clara, CA, USA). Mobile phase was a combination of aqueous phase (40 mmol/L solution of Brij 35 in 7 mmol/L Na_2_HPO_4_) and acetonitrile (70:30, *v*/*v*), and the mobile phase pH was adjusted to 5.5 using *o*-phosphoric acid. The column temperature was set to 36.5 °C, the mobile phase flow rate was set to 1 mL/min, and the detection was performed at the following wavelengths: 220 nm, 240 nm, and 254 nm. All tested compounds were dissolved in DMSO (0.1 mg/mL), diluted with acetonitrile to give final solutions (0.01 mg/mL), and then injected in duplicates. Retention factors (**k**) were calculated according to Equation (1). Column dead time (t_0_) was the retention time of DMSO at 220 nm, while t_R_ was the retention time of a tested compound.

**k** = (t_R_ − t_0_)/t_0_ (1)

### 3.3. PAMPA Test (Parallel Artificial Membrane Permeability Assay)

The phosphate buffer solution was prepared by dissolving 0.5292 g of NaH_2_PO_4_ × H_2_O and 0.0234 g of Na_2_HPO_4_ in purified water (200 mL). The phosphate buffer was divided into two portions, and the pH was adjusted to pH 5.5 and pH 7.4 using *o*-phosphoric acid. The donor solutions were prepared by diluting the stock solutions (5 mM DMSO solutions) with the buffer pH 5.5 to obtain the final concentration of 0.05 mM (the DMSO concentration in the donor solutions was 1%), while the acceptor solution was prepared as a 1% DMSO solution in the buffer pH 7.4. The use of DMSO as a cosolvent in PAMPA is necessary in cases of low-soluble compounds. It is usually used in concentrations up to 5% [25,55,56,57]. Bujard et al. proved the membrane integrity using ethidium bromide, which does not cross the membrane, as well as by electrical resistance measurement, in the experiment where 1% of DMSO was used as a cosolvent [25,57]. In another assay, Petit et al. used 5% of DMSO as a cosolvent and proved membrane integrity using sulfasalazine, which is known to be a non-permeant compound, and testosterone, which is known to be a high permeant compound [55].

The PAMPA test was performed according to the literature procedure using a 96-well microtiter polycarbonate filter plate (Merck Millipore, Carrigtwohill, Ireland) that was impregnated with 15 µL of a hexadecane/hexane (5:95, *v*/*v*) solution [52]. Donor solutions (300 µL per well) were transferred into each well of the donor plate. Acceptor solution was transferred into each well (300 µL per well) of the acceptor plate (MSSACCEPTOR, Millipore, Burlington, MA, USA) and covered by the donor plate to create a PAMPA sandwich. The system was incubated at room temperature for 5 h at a vibratory mixer. After incubation, the PAMPA sandwich was dissociated, and concentrations of all tested compounds in each well, as well as in the starting solutions, were determined using the HPLC method (Section 3.3.1).

#### 3.3.1. HPLC Method

The concentrations of tested compounds in the starting solutions, as well as in the donor and acceptor sections after incubation, were determined by the HPLC method on the Dionex Ultimate 3000 (Thermo Fisher Scientific, Waltham, MA, USA) equipped with a quaternary pump, autosampler, and DAD detector using a ZORBAX Eclipse XDB-C18 4.6 × 150 mm, 5 µm column (Agilent Technologies, Santa Clara, CA, USA). The mobile phase was a combination of acetonitrile and phosphate buffer pH 7.4 (60:40, *v*/*v* for compounds **1**, **2**, **3**, and **12**; 70:30, *v*/*v* for compound **11**; and 30:70, *v*/*v* for NSAIDs). The column temperature was set to 25 °C, the mobile phase flow rate was 1 mL/min, and the detection was performed at the following wavelengths: 220 nm, 240 nm, and 254 nm.

#### 3.3.2. Calculation of PAMPA Parameters

The PAMPA parameters ***R*** (membrane retention) and ***P_e_*** (effective passive permeability value) were calculated using Equations (2) and (3) [23,55].

(2) $$R=1-\frac{C_{D}(t)+C_{A}(t)}{C_{D}(0)}$$

(3) $$P_{e}=-0.00036907\cdot \log\;[1-(\frac{2}{1-R})\frac{C_{A}(t)}{C_{D}(0)}]$$

*R* represents the retention, which is defined as the fraction of the tested compound retained in the PAMPA membrane (filters and plate materials), *C_D_*(*t*) is the concentration of a tested compound in a donor compartment after incubation time t, *C_A_*(*t*) is the concentration of a tested compound in an acceptor compartment after incubation time *t*, while *C_D_*(0) represents the concentration of a tested compound in a donor compartment at the beginning of the incubation (starting solution).

### 3.4. HPAC—Chromatographic Conditions

HPAC analysis was performed on an Agilent 1200 Series chromatograph (Agilent Technologies, Santa Clara, CA, USA). The retention behavior of selected compounds was investigated on CHIRALPAK^®^ HSA column 150 mm × 4 mm I.D. packed with HSA chemically bound to silica particles size of 5 μm (Daicel Corporation, Illkirch-Graffenstaden, France). The mobile phase consisted of ammonium acetate buffer (pH 7.0; 0.01 M) and acetonitrile (85:15, *v*/*v*). The flow rate was 0.6 mL/min, the temperature was set to 25 °C, and the detection of tested compounds was carried out at 283 nm. Tested compounds were dissolved in DMSO (at a concentration of 1 mg/mL), further diluted with the mobile phase to a final concentration of 0.05 mg/mL, and analyzed in triplicate. The HPAC conditions were selected not only with the aim of achieving satisfactory retention characteristics of all synthesized compounds but also taking into account recommendations of a column manufacturer. Retention factors were calculated using Equation (1), where t_0_ (column dead time) was the retention time of DMSO at 220 nm.

### 3.5. Differential Pulse Voltammetry (DPV)

The voltammetric measurements were carried out with the µAutolab analyzer (EcoChemie, Utrecht, The Netherlands). A three-electrode system with a GCE working electrode, an Ag/AgCl reference electrode, and a Pt-auxiliary electrode was used. Before each experiment, the GCE was manually polished with an aqueous slurry of Al_2_O_3_ powder (particle size of 0.05 μm) on a smooth polishing pad, sonicated in deionized water, and then treated in absolute ethanol. Phosphate buffer saline (PBS) pH 7.4 was used as the supporting electrolyte. The experiments were carried out with deaerated PBS buffer. A stock solution of HSA in PBS at a concentration of 0.1 mmol/L was used. The electrochemical behavior of all tested compounds (compounds **1**, **2**, **3**, **11**, and **12**, indomethacin, flurbiprofen, diclofenac, ibuprofen, and naproxen) on GCE was investigated using DPV in the range from 0.0 V to +1.6 V, with the following experimental conditions: potential change increment: 0.005 V, amplitude: 0.005 V, pulse modulation time: 0.05 s, and duration of potential pulse period: 1 s. The interaction between tested compounds and HSA was examined in PBS solution, using solutions of tested compounds in a concentration of 0.05 mmol/L and HSA solution with a concentration in the range of 0.05–10 μmol/L.

The percentage of free compound (Equation (4)) was calculated from the ratio of the current intensity (*I_INT_*) for a given compound concentration (0.05 mmol/L) in the presence of albumin to the signal obtained at the same compound concentration only in the presence of the supporting electrolyte (PBS buffer) (*I_C_*). The percentage of bound drug was calculated using the value obtained for the % free compound according to equation (Equation (5)) [33]:

(4) $$\%\;free\;compound=\frac{I_{INT}}{I_{C}}\cdot 100$$

(5) $$\%\;bond\;compound=1-\%\;free\;compound=\frac{I_{C}-I_{INT}}{I_{C}}\;\cdot 100$$

### 3.6. Stability in Acidic Medium and Solubility

Acidic medium was simulated by a 0.01 M HCl solution, which was prepared by diluting 0.1 M HCl with distilled water. Stock solutions of tested compounds (5 mM DMSO solutions) were diluted with the medium to obtain a concentration of 0.05 mM. Tested compounds were investigated immediately after preparation (t_0_) and after a defined incubation time (t) by injecting these solutions into the HPLC chromatograph in duplicates (the HPLC method used was the same as described in Section 3.3.1). The difference between peak areas at t_0_ and t time points and the eventual occurrence of additional chromatographic peaks after incubation were analyzed.

Solubility of tested compounds was investigated at room temperature. Compounds were weighed on a scale (5 mg each) and transferred to flasks. In a stepwise procedure, increasing volumes of distilled water were added, and after each addition of an amount of water, the mixture was placed on an ultrasound bath for 5 min and visually inspected for undissolved parts of the sample. When no dissolved particles could be observed, the mixture was transferred into a glass cylinder, and opalescence was compared with water. If the mixture was not more opalescent than the solvent, the solvation was considered to be achieved. Solubility of a compound is defined according to the amount of solvent required to dissolve 1 mg [54].

### 3.7. Microsomal Stability

Microsomal stability of tested compounds was investigated using CD-1 mouse liver microsomes, according to the protocol described by Knights et al., using propranolol as a positive control [58]. Stock solutions of all tested compounds (5 mM DMSO solutions) were diluted with 0.1 M potassium phosphate buffer pH 7.4 to obtain 0.1 mM solutions, which were further incubated according to the protocol. In order to activate both the 1st and 2nd phases of metabolism, NADPH and UDP-GlcUA were used as cofactors. Tested compounds were incubated at 37 °C for 30 min, and the incubation was quenched immediately after the addition of cofactors (t_0_) and after the defined incubation time (t) by mixing 100 µL of corresponding incubation mixtures with 200 µL of cold acetonitrile. All compounds were tested in duplicates, and their concentrations were determined using the HPLC-MS/MS method (Section 3.7.1).

#### 3.7.1. HPLC-MS/MS Method

The HPLC-MS/MS analysis was performed on the UHPLC chromatograph ACELLA (Thermo Fisher Scientific Inc., Madison, WI, USA), coupled to a triple quadrupole mass spectrometer TSQ Quantum Access MAX (Thermo Fisher Scientific Inc., Madison, WI, USA) with a heated electrospray ionization (HESI) interface. The column used was Zorbax Extend C18 (150 mm × 4.6 mm, 5 µm; Agilent Technologies), which was thermostated at 25 °C. The mobile phase consisted of acetonitrile and 0.1% formic acid (60:40, *v*/*v*), with a flow rate of 500 µL/min. The injection volume was 10 µL. A positive SRM mode was used for the detection and quantification of compound **1** (*m*/*z*: 388.1–312.2), compound **2** (*m*/*z*: 275.3–199.2), compound **3** (*m*/*z*: 326.0–214.0), compound **11** (*m*/*z*: 236.2–119.3), compound **12** (*m*/*z*: 260.2–170.2), and propranolol (*m*/*z*: 260.2–116.2). A positive SIM mode was used for the detection and quantification of indomethacin (*m*/*z*: 358.1), while a negative SIM mode was used for diclofenac (*m*/*z*: 294.1).

### 3.8. Statistical Analysis

The significance of differences among obtained results was assessed by the Student’s *t*-test (Paired Two Sample for Means).

## 4. Conclusions

Compounds **1**, **2**, **3**, **11**, and **12** (N-hydroxyurea and hydroxamic acid derivatives and analogs of commercially available NSAIDs indomethacin, flurbiprofen, diclofenac, ibuprofen, and naproxen, respectively) were subjected to in vitro evaluation of selected pharmacokinetic properties. Indomethacin, flurbiprofen, diclofenac, ibuprofen, and naproxen (parent NSAIDs with COX inhibitory activity) were used as standards for comparison. Two methods were employed to evaluate passive gastrointestinal absorption (BMC and PAMPA). The findings were fairly consistent, leading to the conclusion that the introduction of N-hydroxyurea and hydroxamic acid groups into the NSAID structure lowers passive gastrointestinal absorption. Given that oral drug administration is the most preferred route, structural modifications of compounds **1**, **2**, **3**, **11**, and **12** that increase lipophilicity (such as the addition of an aromatic ring or alkylation of the nitrogen in the N-hydroxyurea group) could be considered to enhance their passive gastrointestinal absorption. Binding affinity for HSA was also evaluated using two in vitro methods (HPAC and DPV), with results that were also quite consistent. The general conclusion was the same as for the passive gastrointestinal absorption—incorporation of the N-hydroxyurea and hydroxamic acid groups into the NSAIDs structure decreases binding affinity for HSA, most likely due to the decreased acidity in comparison to parent NSAIDs. Reduced binding to HSA may potentially decrease interactions with other drugs. Considering lower expected HSA binding and higher lipophilicity of compounds **1**, **2**, **3**, **11**, and **12** compared to corresponding NSAIDs, it can be expected that the volume of distribution of compounds **1**, **2**, **3**, **11**, and **12** will be higher. In order to get better insight into the influence of these chemical modifications on observed pharmacokinetic parameters, further computational analyses should be performed. Compounds **1**, **2**, **3**, **11**, and **12** showed higher microsomal instability in comparison to standard NSAIDs indomethacin and diclofenac, which indicates their higher susceptibility to liver metabolism.

## Figures and Tables

**Table 1:** Retention factors (**k**) of tested compounds.

Compound	k
**1**	9.50 ± 0.01 (*p* = 0.0156) ^a^
**2**	5.55 ± 0.01 (*p* = 0.0383)
**3**	9.33 ± 0.03 (*p* = 0.0056)
**11**	3.03 ± 0.01 (*p* = 0.0003)
**12**	3.02 ± 0.00 (*p* = 0.0270)
**Indomethacin**	10.20 ± 0.03
**Flurbiprofen**	7.14 ± 0.02
**Diclofenac**	8.25 ± 0.00
**Ibuprofen**	13.36 ± 0.00
**Naproxen**	5.29 ± 0.27

^a^ *p* values were calculated for each pair tested compound—corresponding standard.

**Table 2:** PAMPA permeability coefficients of tested compounds.

Compound	Pe	–logPe
**1**	4.29 × 10^−5^ ± 7.85 × 10^−6^	4.37 ± 0.08
**2**	1.76 × 10^−5^ ± 0.96 × 10^−6^	4.76 ± 0.02
**3**	7.73 × 10^−5^ ± 4.19 × 10^−6^	4.11 ± 0.02
**11**	14.59 × 10^−5^ ± 15.67 × 10^−6^	3.84 ± 0.05
**12**	15.70 × 10^−5^ ± 13.41 × 10^−6^	3.81 ± 0.04
**Indomethacin**	38.63 × 10^−5^ ± 45.00 × 10^−6^6	3.41 ± 0.05
**Flurbiprofen ^a^**	>40.00 × 10^−5^	<3.40
**Diclofenac ^a^**	>40.00 × 10^−5^	<3.40
**Ibuprofen ^a^**	>40.00 × 10^−5^	<3.40
**Naproxen**	34.67 × 10^−5^ ± 50.83 × 10^−6^	3.46 ± 0.06

^a^ Due to pronouncedly high ratios between concentrations in acceptor compartments after incubation and concentrations in starting solutions (>52%), **Pe** and **–logPe** could not be calculated, so these values were denoted as >40.00 × 10^−5^ and <3.40, respectively.

**Table 3:** Retention factors (**logk**) of tested compounds.

Compound	Retention Factors (logk)
**1**	9.54 ± 0.29
**2**	7.45 ± 0.02
**3**	8.24 ± 0.01
**11**	5.81 ± 0.04 ^a^
**12**	2.03 ± 0.02
**Indomethacin**	>70
**Flurbiprofen**	11.03 ± 0.02
**Diclofenac**	>70
**Ibuprofen**	>70
**Naproxen**	>70

^a^ average retention time of two close peaks since it is a mixture of diastereoizomers.

**Table 4:** DPV peak potential shifts and the percentage of bound drug after the interaction of compounds with HSA.

Compound	ΔE (mV)	% Bound Drug
**1**	34	73.3
**2**	30	80.2
**3**	29	80.2
**11**	7	70.8
**12**	13	70.4
**Indomethacin**	70	94.3
**Flurbiprofen**	Electrochemically inactive	Electrochemically inactive
**Diclofenac**	54	80.5
**Ibuprofen**	Electrochemically inactive	Electrochemically inactive
**Naproxen**	168	88.4

**Table 5:** Chromatographic peak areas of tested compounds in acidic medium (pH = 2).

Compound	Peak Area (t_0_)	Peak Area (t)	The Decrease in Peak Area (%)
**1**	8.28 ± 0.01	8.27 ± 0.00	0.07 (*p* = 0.2839) ^a^
**2**	6.57 ± 0.12	6.55 ± 0.07	0.34 (*p* = 0.4501)
**3**	6.42 ± 0.11	6.27 ± 0.10	2.33 (*p* = 0.0159)
**11**	11.01 ± 0.02	10.83 ± 0.03	1.63 (*p* = 0.073)
**12**	48.66 ± 0.49	48.54 ± 0.39	0.24 (*p* = 0.4399)

^a^ *p* values were calculated for each pair t_0_ − t.

**Table 6:** Chromatographic peak areas and the decrease in peak area after incubation of tested compounds.

Compound	Peak Area, t_0_ (×10^3^)	Peak Area, t (×10^3^)	The Decrease in Peak Area (%)
**1**	131.5 ± 16.8	60.5 ± 6.4	53.98 (*p* = 0.0328) ^a^
**2**	16.5 ± 3.1	9.6 ± 3.0	41.65 (*p* = 0.0034)
**3**	1033.6 ± 42.5	455.5 ± 8.9	55.93 (*p* = 0.0200)
**11**	60.0 ± 1.4	44.6 ± 1.9	25.07 (*p* = 0.0499)
**12**	43.9 ± 3.2	18.2 ± 0.1	58.44 (*p* = 0.0273)
**Indomethacin**	241.9 ± 2.5	206.9 ± 9.0	14.47 (*p* = 0.0419)
**Diclofenac**	126.2 ± 2.8	99.7 ± 5.3	20.99 (*p* = 0. 0213)
**Propranolol**	268.5 ± 17.0	155.7 ± 17.9	42.03 (*p* = 0.0017)

^a^ *p* values were calculated for each pair t_0_ − t.

## Data Availability

Data are contained within the article.
